# Prevalence of self-harm across urgent and emergency care settings among young people and factors associated with reattendance: protocol for a prospective cohort study

**DOI:** 10.1136/bmjopen-2025-114062

**Published:** 2026-01-21

**Authors:** Daniel Romeu, Samuel Relton, Christopher Burton, Annabel Crum, Emily V Chambers, David Cottrell, Elspeth Guthrie

**Affiliations:** 1 Leeds Institute of Health Sciences, University of Leeds, Leeds, UK; 2 University of Sheffield, Sheffield, UK

**Keywords:** Suicide & self-harm, Emergency Departments, Paediatric A&E and ambulatory care, Health Services, Child & adolescent psychiatry, MENTAL HEALTH

## Abstract

**Abstract:**

**Introduction:**

Self-harm represents a significant public health concern and is a common reason for contact with urgent and emergency care (UEC) services among young people. Although young people frequently interact with multiple components of the urgent care system following self-harm, there is limited system-level evidence describing patterns of service use, transitions between services and repeat emergency department (ED) attendance. An improved understanding of how young people use UEC services after self-harm is needed to inform the design of more effective and appropriate care pathways.

**Methods and analysis:**

This protocol describes a prospective cohort study using an extract from the Centre for URgent and Emergency care research database (CUREd+) research database, which comprises routinely collected, linked healthcare data from the National Health Service 111 (NHS 111), ambulance services, urgent care centres, walk-in centres and EDs across Yorkshire and the Humber, England. The study population will include young people aged ≤25 years presenting to UEC services between April 2019 and March 2022 with self-harm coded as the reason for attendance. Analyses will describe the prevalence of self-harm presentations across UEC settings, quantify the proportion of NHS 111 and ambulance contacts resulting in ED attendance within 24 hours and examine factors associated with ED reattendance at 3 and 12 months. Mixed-effects logistic regression models will be used to account for repeated attendances, confounding variables and temporal variation, including changes related to the COVID-19 pandemic. Anticipated analysis period: January 2026–January 2027.

**Ethics and dissemination:**

Ethical approval has been granted by the University of Leeds (MREC 22-079 Amd1) and the University of Sheffield (Ref 068194). The CUREd+ research database operates under Research Ethics Committee approval (23/YH/0079) and Confidentiality Advisory Group approval (18/CAG/0126). Individual consent is not required as all data are pseudonymised at source. Findings will be disseminated through peer-reviewed publications, conference presentations and public-facing outputs coproduced with patient and public involvement groups.

**Strengths and limitations of this study**

- This study will use the Centre for URgent and Emergency care research database (CUREd+), providing routinely collected, pseudonymised records on over 20 million contacts, enabling population-level coverage and pathway mapping.
- The prospective cohort design and linkage of repeated attendances will allow identification of risk factors for reattendance and care trajectories.
- Young people and carers have been actively involved in shaping study aims, variables of interest and dissemination plans through two dedicated patient and public involvement groups.
- Analyses will be limited to routinely collected data, so information on intent behind self-harm presentations, severity and psychosocial factors may be incomplete, and findings may not capture presentations outside of National Health Service urgent and emergency services.

## Background

 Self-harm is defined as any purposeful act of self-injury or self-poisoning, regardless of the underlying intent.^1^ It is a complex spectrum of behaviours with multiple causes and functions.^2^ Self-harm is strongly associated with adverse outcomes, including mental illness, poorer educational development and premature death.^3^ Importantly, it is strongly associated with future suicide,^4^ which remains a leading cause of death among young people in the UK.

Self-harm is a common reason for contact with urgent and emergency care (UEC) services. More than an estimated 220 000 hospital episodes in England each year are attributed to self-harm,^5^ and such presentations are rising.^6^ Crisis presentations to emergency departments (EDs) have become the default route to accessing timely mental healthcare, largely due to a lack of community-based alternatives to hospitals for self-harm, which is reflected in first-hand accounts of young people.^7^ This adds to wider pressures on the National Health Service (NHS), which faces well-documented challenges in providing timely and appropriate urgent mental healthcare.^8^

Ambulance services are frequently the first point of contact with emergency services. Around 11% of calls for people over 15 years relate to self-harm, and more than half result in conveyance to the ED.^9^ However, many patients do not require acute medical treatment, and hospital attendance may be distressing or counterproductive. Ambulance clinicians report that system failures leave them with few alternatives to ED conveyance.^10^ Training in mental healthcare for paramedics is limited, with only 15% reporting feeling prepared to manage self-harm presentations.^11^

Within EDs, staff also face significant challenges. A meta-synthesis of UK hospital staff perspectives found feelings of fear and being underskilled when caring for people who self-harm.^12^ Negative experiences are commonly reported by patients, including stigmatising comments or refusal of care.^13^ Such encounters may exacerbate distress, increase the risk of repeat self-harm^14^ and reduce willingness to seek help in the future.^15^

Emergency mental healthcare has been recognised as a national priority. The National Institute for Health and Care Excellence (NICE) guideline on self-harm describes it as ‘everyone’s business’ and highlights the urgent need for research on effective models of care for young people.^1^ In response, NHS England has published new implementation guidance on emergency mental health services for children and young people, emphasising prevention and community-based alternatives to admission.^16^

The UK Government’s 10 Year Health Plan (2025) commits up to £120 million to establish dedicated mental health EDs and expand crisis response pilots, including mental health response vehicles.^17^ This reflects a policy shift towards ensuring that patients receive specialist support in appropriate settings, rather than defaulting to general EDs. These reforms acknowledge the mismatch between current service provision and the needs of people experiencing self-harm or acute mental distress. Despite this momentum, there is little evidence describing how young people actually navigate UEC services after self-harm, and which factors influence repeated service use.

Routinely collected NHS data offer an opportunity to address this gap. Understanding patterns of self-harm presentation across the full UEC pathway (NHS 111, ambulance, urgent care centres, walk-in centres and EDs) is critical to informing service planning and provision. Evidence on repeat presentations will also help identify groups at greatest risk and clarify where early intervention may reduce future harm.

### Aims and objectives

This study aims to describe the epidemiology of self-harm presentations to UEC services among young people in Yorkshire and the Humber, England. The specific objectives are to:

1. Determine the prevalence of self-harm as the reason for presentation across the UEC pathway among young people (≤25 years).
2. Establish the proportion of NHS 111 and ambulance calls leading to an ED attendance within 24 hours for self-harm.
3. Identify factors associated with repeat ED presentations for self-harm at 3 and 12 months after an index episode.

By clarifying how young people use UEC services after self-harm, this study will generate evidence directly relevant to national policy priorities. Findings will support commissioning decisions to ensure that emergency mental healthcare is delivered in the right place, at the right time and by appropriately skilled professionals.

## Methods and analysis

### Study design and setting

This protocol describes a longitudinal cohort study using routinely collected NHS data from the Centre for URgent and Emergency care research database (CUREd+) research database. The study will examine patterns of UEC use by young people (≤25 years) following self-harm presentations in Yorkshire and the Humber, a region with 5.4 million residents (10% of the English population) and wide social and geographical diversity.^18^ This protocol was developed in accordance with the Strengthening the Reporting of Observational Studies in Epidemiology (STROBE) guidelines for cohort studies.^19^

### Data source

The CUREd+ research database (https://sheffield.ac.uk/data-connect/data-assets/cured-research-database) is hosted by the University of Sheffield and collates linked routine data from UEC providers in England. The CUREd+ extract created for this study covers Yorkshire and the Humber and includes data from the Yorkshire Ambulance Service (YAS), NHS 111, acute NHS trusts and 18 EDs. The extract represents over 10% of England’s population.^20^ Data are available from April 2011 to March 2023. For the present study, the analysis period is April 2019 to March 2022, allowing analysis of service use before, during and after COVID-19 pandemic lockdown restrictions.

The CUREd+ database examines patient journeys through different health services by linking secondary care datasets using personal information. Data are pseudonymised at source; no identifiable information is held in the research database. Linkage is achieved through encrypted identifiers, enabling patient pathways to be tracked across services. Data extracts are available on request by researchers subject to appropriate permissions, and access is restricted to trained personnel at the University of Sheffield.

### Participants and eligibility

We will include all individuals aged ≤25 years (at the time of the index presentation) who presented to a UEC service between April 2019 and March 2022 with self-harm coded as the reason for attendance. This age range was selected because 75% of mental health disorders begin before the age of 25 years,^21^ and to align with the conventional upper age limit for young people in self-harm studies.^22^ For this study, the index event is defined as the first eligible presentation within the study period. Participants may contribute multiple attendances for follow-up analyses. The final cohort size will depend on filtering but is anticipated to include approximately 16 000 self-harm presentations, based on national estimates,^9 23^ although this will depend on data filtering.

### Determining self-harm events

Accurate identification of self-harm events in routine data is challenging. Coding practices in emergency settings are variable and may underestimate true prevalence.^24^ To address this, comprehensive self-harm code lists have been developed with input from senior NHS psychiatrists, a data scientist with experience of the CUREd dataset (a smaller, regional 2011–2017 predecessor to CUREd+ also hosted by the University of Sheffield) and the CUREd+ data management team. International Classification of Diseases 10th Revision (ICD-10) codes were adapted from published self-harm code lists; Systematised NOmenclature of MEDicine Clinical Terms (SNOMED CT) codes were generated by reverse mapping from ICD-10; service-specific codes (including YAS ambulance codes) were selected through review of established code lists. All candidate codes were reviewed by the multidisciplinary team. This approach seeks to balance sensitivity (capturing as many true cases as possible) with specificity (minimising false positives).

### Variables

The primary outcome of this study is the prevalence of self-harm as the reason for presentation to different components of the UEC pathway (NHS 111, ambulances, walk-in centres, urgent care centres, EDs). Secondary outcomes include the proportion of NHS 111 and ambulance calls leading to ED attendance within 24 hours for self-harm, and factors associated with repeat ED presentations for self-harm at 3 and 12 months postindex presentation. Covariates include demographic characteristics (age, sex, ethnicity, deprivation index), comorbidities and calendar year.

In addition to CUREd+ UEC data, linked information from the Civil Registrations of Deaths and Medicines Dispensed in Primary Care (MDPC) datasets will be used. Deaths data will be used to establish which patients died during follow-up, supporting accurate estimation of reattendance rates and contextual interpretation of mortality after self-harm. The MDPC dataset will be included to provide intelligence on medicine safety and effectiveness, consistent with its approved use under the NHS England data sharing agreement for the CUREd+ database. Analyses using MDPC will focus on exploring psychotropic medication-prescribing patterns preceding and following self-harm presentations and on identifying medicines associated with repeat attendances or adverse outcomes. These datasets will strengthen the study’s ability to examine outcomes relevant to both service delivery and medication safety.

### Data cleaning and preparation

Prior to analysis, data will undergo systematic cleaning to ensure accuracy, consistency and utility. This will include:

- Deduplication: identifying and removing duplicate records across services using encrypted patient identifiers.
- Validation of time stamps: ensuring chronological consistency across linked service records, for example, NHS 111 calls preceding ED attendance.
- Standardisation of variables: harmonising coding formats across services to ensure compatibility and meaningful analysis.
- Handling missing data: descriptive statistics will be used to assess the extent and patterns of data missingness; missing data will be handled appropriately (eg, using multiple imputation), particularly for key covariates such as ethnicity and indices of deprivation.
- Outlier detection: extreme values (such as implausible age or time intervals) will be flagged and reviewed through consultation with the CUREd+ data management team and removed where appropriate.

All data cleaning procedures will be documented and reproducible, with code available on request to improve transparency and replicability.

### Statistical methods

We will first describe the prevalence of self-harm presentations across UEC services, stratified by age and sex. Care pathways will then be examined, identifying the proportion of NHS 111 and ambulance calls that result in ED attendance within 24 hours for self-harm.

For predictors of ED reattendance, we will use mixed-effects logistic regression. Fixed effects will include demographic and clinical variables, with non-linear terms where appropriate (eg, age). A random effect for participant ID will account for repeated attendances. Confounding will be addressed by adjusting for age, sex, comorbidities and calendar year.

To reduce the risk of overfitting, model size will be modest and focused on key demographic and clinical covariates (<40 parameters are anticipated). With an anticipated sample of approximately 16 000 index self-harm presentations, this will equate to at least 400 events per parameter, far exceeding the commonly cited minimum threshold of 10–20 events per parameter for logistic regression.^25^ Non-linear terms will only be introduced where there is a strong justification, and model parsimony will be prioritised.

For sensitivity analysis, analyses will be conducted separately for each study year (2019/2020, 2020/2021, 2021/2022) to account for potential changes during the COVID-19 pandemic. Statistical analyses will be performed in R (using the lme4 package). Additional sensitivity analyses may be undertaken to exclude pandemic years or to stratify by age groups, depending on data trends.

### Addressing bias

Potential sources of bias and mitigation strategies include:

- Misclassification (undercoding or inconsistent coding of self-harm) will be mitigated through comprehensive, expert-reviewed code lists.
- Missing data will be handled through descriptive reporting and, where feasible, multiple imputation or other appropriate methods.
- Confounding will be controlled using multivariable regression.
- Although the use of a large regional dataset reduces selection bias, it cannot be eliminated. Presentations may be miscoded or missed, particularly if individuals do not access UEC services. Findings will be limited to those who use NHS services in Yorkshire and the Humber and may not capture those who self-harm but do not seek help from healthcare services. These factors will be acknowledged during data interpretation.

### Patient and public involvement

Patient and public involvement (PPI) has been embedded in the design and planned conduct of this study. Early engagement with five mental health PPI groups (30 members) confirmed the importance of this research. We convened two lived experience advisory groups, a Young People’s Advisory Group (five members aged 14–25) and a Parents and Carers’ Advisory Group (four members). Both groups have contributed to decisions around the study aims and variables of interest. Meetings with the PPI groups will be held every 4 months during the project to coproduce interim analyses and accessible dissemination (including animated videos and other public-facing outputs). PPI contributors will be offered training and support for analysis and writing tasks and will be reimbursed in line with the National Institute for Health and Care Research (NIHR) guidance. PPI representatives will be acknowledged in publications and coauthorship will be offered where contributions meet journal criteria.

## Discussion

### Anticipated outputs

This study is designed to generate a contemporary, data-driven overview of how young people (≤25 years) use and navigate UEC services after self-harm in Yorkshire and the Humber. Using linked data from the CUREd+ research database, it will examine the prevalence and pathways of self-harm presentations across NHS 111, ambulance services, urgent care centres and EDs. Statistical modelling will examine factors associated with reattendance at the ED at 3 and 12 months postindex presentation. Outputs will include tabular summaries quantifying pathway progression (including proportion of ambulance calls leading to ED attendance) and regression analyses of predictors of reattendance.

Beyond descriptive statistics, findings will support the development of programme theories within the wider Emergency Care After Self-Harm (EmCASH) study, a realist, synthesis and mixed-methods evaluation of emergency care for young people who self-harm.^26^ Insights from routine data will contribute to explanatory models of how and why emergency services produce intended and unintended outcomes, which will be refined through qualitative research with young people and their caregivers. The present study forms a critical foundation for theory-driven improvements in emergency mental healthcare.

### Interpretation and contribution

Self-harm is a major and rising cause of UEC use, yet current services often fail to meet the needs of young people. Existing evidence demonstrates both the scale of the problem^5^ and the shortcomings of current service pathways, including limited mental health training for frontline staff,^27^ stigma within EDs^13^ and geographical variation in access to alternatives to hospitals.^7^

By mapping care pathways across multiple entry points to the UEC system, this study will provide novel evidence on how young people engage with services following self-harm and where bottlenecks or inefficiencies occur. In doing so, it directly addresses national priorities. The 10 Year Health Plan for England commits to new models of emergency mental health provision, including specialist mental health EDs and crisis response vehicles,^17^ while NICE guidelines emphasise the need for research on effective care for young people who self-harm.^1^ The current crisis in emergency mental healthcare reflects a fundamental misalignment between patient needs and system design.^8^ Evidence from this study will be critical for informing service configuration in line with these policy commitments.

Findings will also have immediate clinical implications. By identifying predictors of repeat self-harm presentations, the study may highlight groups at greatest risk of poor outcomes, providing an evidence base to support targeted interventions. More broadly, it will clarify where timely alternatives to ED attendance could improve patient experience while alleviating pressures on overstretched services.

### Strengths and limitations

A key strength of this study is its use of an extract from the CUREd+ database, which provides linked, patient-level data across the full UEC pathway for a large and diverse regional population. This enables examination of service use at scale, capturing interactions between NHS 111, ambulance services, urgent care centres and EDs, a perspective rarely available in studies of self-harm. The longitudinal design allows examination of outcomes at 3 and 12 months, generating insights into recurrent service use.

However, there are important limitations to consider. Importantly, the identification of self-harm presentations relies on routine clinical coding, which is known to be inconsistent and prone to under-recording. Coding practices in emergency settings are variable and influenced by clinical uncertainty, stigma and organisational pressures.^24^ This study seeks to mitigate these challenges through the development of comprehensive code lists, informed by existing published lists and reviewed by psychiatrists and a statistician with expertise in CUREd+ data. However, misclassification remains possible and may influence prevalence estimates. Second, missing or incomplete data may limit the accuracy of covariates such as comorbidities or socioeconomic status. Strategies such as multiple imputation will be considered, but residual bias cannot be excluded. Third, as an observational study, causal inference is limited; associations between service pathways and outcomes must be interpreted with caution. Finally, the study is geographically restricted to Yorkshire and the Humber. While this is a large and sociodemographically diverse region representing over 10% of the English population,^20^ findings may not fully generalise to other regions with different service configurations or population profiles.

### Generalisability and impact

Our findings are likely to have national relevance. Yorkshire and the Humber encompasses a range of urban and rural settings, high and low deprivation and ethnically diverse populations, reflecting many of the challenges faced across England. The study’s integration into the EmCASH programme further strengthens its generalisability by linking quantitative findings to qualitative accounts from young people and caregivers across diverse settings.

At the service level, findings will inform commissioning decisions and highlight opportunities to improve care pathways, reduce unnecessary conveyance to EDs and enhance patient experience. At the policy level, outputs will support NHS England and Integrated Care Boards in implementing the 10 Year Health Plan and NICE guidance. By providing contemporary, real-world evidence, the study has potential to shape the design of new emergency mental health services, including the scaling up of crisis response vehicles and the development of dedicated mental health EDs.

### Ethics and dissemination

Ethical approval has been granted by the University of Leeds and the University of Sheffield, and the CUREd+ database operates under NHS Research Ethics Committee and Confidentiality Advisory Group approvals. Individual consent is not required as data are pseudonymised at source. All research procedures will be conducted in accordance with the Declaration of Helsinki.^28^

Study findings will be disseminated through multiple channels to maximise reach and impact. Academic outputs will include peer-reviewed publications and presentations at national and international conferences. Reports will be submitted to the funder (NIHR) and shared with stakeholders including NHS England, Integrated Care Boards and service providers. To ensure accessibility, findings will be summarised in patient and public-facing formats, supported by the EmCASH project website and blogs. Engagement with patient and public involvement and engagement groups will guide the design of dissemination materials.

In line with the wider EmCASH programme, findings from this study will be used to refine realist programme theories, which will subsequently be tested and developed through qualitative research with young people and their caregivers. This study therefore provides immediate outputs and contributes to a broader effort to develop evidence-based, theoretically informed models of emergency care for young people who self-harm.
