# Supplementary material for: Prevalence of self-harm across urgent and emergency care settings among young people and factors associated with reattendance: protocol for a prospective cohort study
Source: BMJ Open. 2026 Jan 21;16(1):e114062. doi: 10.1136/bmjopen-2025-114062 (PMC12829348; doi:10.1136/bmjopen-2025-114062)
Supplement: Supplementary file 1 [file bmjopen-2025-114062.reviewer_comments.pdf]

PEER REVIEW HISTORY

BMJ Open publishes all reviews undertaken for accepted manuscripts. Reviewers are asked to complete a checklist review form and are provided with free text boxes to elaborate on their assessment. These free text comments are reproduced below.

ARTICLE DETAILS

Title (Provisional)

Prevalence of self-harm across urgent and emergency care settings among young people and factors associated with re-attendance: protocol for a prospective cohort study

Authors

Romeu, Daniel; Relton, Samuel David; Burton, Christopher D; Crum, Annabel; Chambers, Emily V; Cottrell, David; Guthrie, Elspeth

---

This article was not externally reviewed at BMJ Open. Protocols that have been independently assessed prior to submission to BMJ Open are usually fast-tracked to publication on the grounds that further substantial changes will not be possible. This independent assessment will usually be external, independent review for both a substantial grant award from a non-commercial or government funder and ethics approval.
